# BEYOND 2 years: durability of metabolic benefits by simplification of complex insulin regimens in type 2 diabetes

**DOI:** 10.1007/s12020-023-03547-9

**Published:** 2023-10-03

**Authors:** Dario Giugliano, Miriam Longo, Lorenzo Scappaticcio, Paola Caruso, Maurizio Gicchino, Michela Petrizzo, Giuseppe Bellastella, Maria Ida Maiorino, Katherine Esposito

**Affiliations:** 1 Department of Advanced Medical and Surgical Sciences, Università della Campania “Luigi Vanvitelli,”, Naples, Italy; 2 Division of Endocrinology and Metabolic Diseases, University Hospital, Università della Campania “Luigi Vanvitelli,”, Naples, Italy

**Keywords:** Type 2 diabetes, Basal bolus insulin regimen, Fixed-ratio combination, Basal insulin plus gliflozin, HbA1c, BEYOND trial

## Abstract

**Purpose:**

To assess the magnitude and durability of the metabolic benefits by simplification of complex insulin treatments in patients with type 2 diabetes inadequately controlled by a full basal-bolus insulin regimen. Herein we report the results of the scheduled 2-year extension of the BEYOND trial.

**Methods:**

Originally, 305 participants with inadequate glycemic control (HbA1c > 7.5%) were randomly assigned to intensification of basal-bolus insulin regimen (*n* = 101), to a fixed-ratio combination (basal insulin + GLP-1RA, *n* = 102), or to an association of basal insulin plus an SGLT-2 inhibitor (gliflo-combo, *n* = 102). The primary efficacy outcome was change from baseline in HbA1c at 24 months assessed by an intention-to-treat analysis. A per-protocol analysis was also performed.

**Results:**

Fifty-five percent of patients completed the study in the two comparison arms. Compared with patients randomized to basal-bolus, patients of the other groups experienced non statistically different reductions in HbA1c level according to either an intention-to-treat analysis (−0.8 ± 1.1%, −0.7 ± 1.1%, and −1.3 ± 1.1%, mean ± SD, fixed-ratio, gliflo-combo and basal bolus, respectively) or per-protocol analysis (−1.2 ± 1.0%, −1.2 ± 1.1%, and −1.3 ± 1.0%, respectively). The final HbA1c level (per protocol) was 7.2 ± 0.8%, 7.3 ± 0.9%, and 7.5 ± 0.9%, respectively (*P* = NS). Treatment satisfaction (DTSQ) increased in both exchange groups, whereas the proportion of patients with hypoglycemia was lower.

**Conclusion:**

Simplification of complex insulin regimen may be a durable option in at least one-half of patients with type 2 diabetes.

**Clinical trial registration:**

Clinical trial registration no. NCT04196231, clinicaltrials.gov.

## Introduction

Given the natural history of type 2 diabetes and the progressive decline in beta-cell function, most patients with the disease will undergo treatment intensification with the aim of preventing or delaying long-term complications [1]. However, many individuals fail to achieve adequate lowering of glycated hemoglobin (HbA1c), even when using complex insulin regimens [2–4]. The American Diabetes Association guidelines highlight the need for personalization of care using shared decision-making and the importance of optimizing quality of life [5].

Simplification reduces the number of insulin injections and adapts the treatment strategy to consider each person’s circumstances [6]. The BEYOND trial [7] evaluated the feasibility of switching individuals with type 2 diabetes and inadequate glycemic control from a basal-bolus insulin regimen to a fixed-ratio combination (FRC) of basal insulin with a GLP-1 RA or a sodium-glucose co-transporter 2 inhibitor (gliflo-combo). After 6 months, similar improvements in HbA1c were reported in both those who were switched to an FRC or to basal insulin with a gliflozin compared to those managed with a basal-bolus regimen, with the added benefit of fewer daily injections, less hypoglycemia, and no weight gain.

We report here the scheduled 2-year extension of BEYOND trial to assess the magnitude and the durability of the metabolic benefits of simplification.

## Materials and methods

### Study design

The three-arm trial design of BEYOND has been described previously [7]. The study protocol was approved by the ethics committee, and all study participants gave written informed consent.

BEYOND was a 6-month, randomized, pragmatic, parallel group, single-center trial that evaluated the efficacy and safety of either FRC or basal insulin plus SGLT-2i to replace a full basal-bolus insulin regimen in participants with type 2 diabetes experiencing inadequate glycemic control (HbA1c > 7.5% [58 mmol/mol]), with or without metformin. The active control group consisted of patients continuing the basal-bolus regimen with their usual diabetes care. Recruitment started in July 2019 and the first part of the trial was completed in 2020 after a follow-up of 6 months. The present report consists of the data obtained up to 30 November 2022, when the last patient competed 2 years after randomization.

Originally, 101 participants were randomly assigned to intensification of basal-bolus regimen, 102 to the FRC group, and 102 to the gliflo-combo group. There were 12 dropouts in the FRC group, 9 in the gliflo-combo group, and none in the basal-bolus group. In the extension phase of the trial, the investigators may suspend the medication for safety reasons or for uncontrolled hyperglycemia (i.e., HbA1c higher than the baseline value in the first assessment). Participants in both the FRC and gliflo-combo groups who withdrew from the study due to lack of treatment efficacy returned to the basal-bolus therapy but were not included in the original basal-bolus group.

### Outcomes

The primary efficacy outcome of the extension trial was change from baseline in HbA1c at 24 months. Secondary outcomes were the proportion of participants with HbA1c < 7.0% (53 mmol/mol) or <7.5% (58 mmol/mol), total daily insulin doses, number of daily injections, percentage of participants with hypoglycemia, changes from baseline in body weight and fasting plasma glucose, and the level of satisfaction (DiabetesTreatment Satisfaction Questionnaire—DTSQ) [8].

To limit clinical inertia, in the extension part of the trial clinicians had to improve glycemic control according to a titration algorithm for basal insulin in all three groups and for prandial insulin in the basal-bolus group (Table 1). Level 1 hypoglycemia was defined as a blood glucose level <70 mg/dL associated with symptoms or signs (sweating, tremor, and tachycardia); level 2 hypoglycemia as a blood glucose level <54 mg/dL; and level 3 (severe) as a blood glucose level <50 mg/dL or needing the assistance of a third party.

**Table 1:** Titration algorithm for recommended rapid insulin analog dose adjustment in the basal-bolus group

Plasma glucose before lunch and dinner and before bedtime (mg/dL)	Adjustment of lispro (U)
≤99 without obvious explanation	−2 UI
100–119 = target	No adjustment
120–139	+2
140–179	+3
≥180	+4

Before breakfast plasma glucose (mg/dL)	Adjustment of basal insulin
<56	−4
56–69	−2
70–79	−1
80–109 = target	No adjustment
110–139	+2
140–179	+4
≥180	+6

Titration algorithm for recommended basal insulin dose adjustment in the three groups

### Statistical analysis

The efficacy population consisted of all participants randomized to study treatment (intention-to-treat). A per-protocol analysis was also performed. Categorical variables are expressed as frequencies and proportions; continuous variables are given as mean ± SD (variables normally distributed) or median and interquartile range (variables not normally distributed). Statistical differences in the primary outcome at 24 months were assessed by ANCOVA with treatment as fixed effect and baseline levels as covariates. The proportions of patients analyzed for secondary outcomes were assessed by contingency tables and χ^2^ test. Differences between baseline parameters and 24 months are assessed by two-sample test for comparisons within groups. The safety population consisted of participants who received at least one dose of randomized study medication. *P* values < 0.05 were deemed statistically significant. We conducted all analyses using SPSS version 26.0 (SPSS Inc., Chicago, IL).

## Results

### Baseline characteristics

**Fig. 1:**
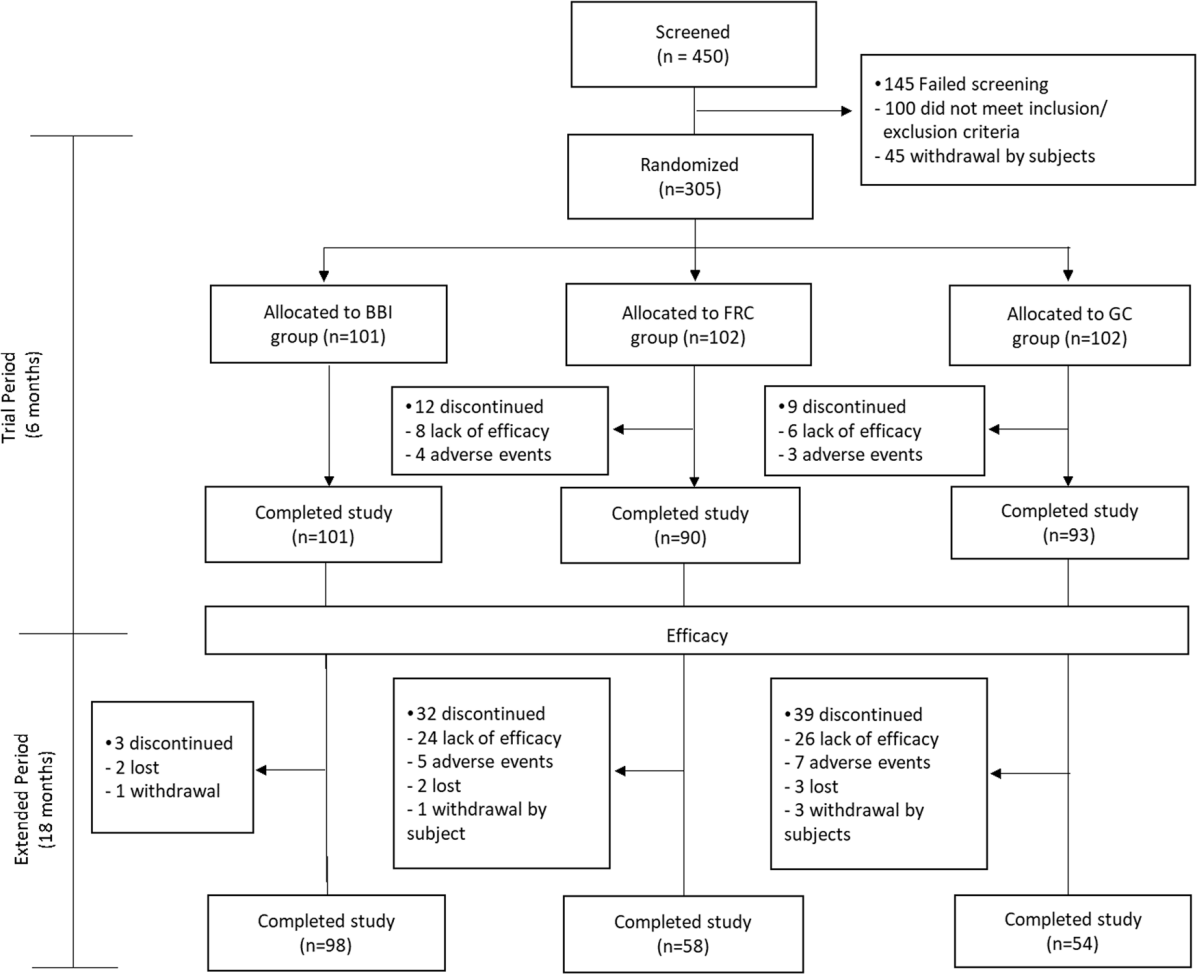
The trial has been completed in 24 months. BBI basal-bolus insulin, FRC fixed-ratio combination (basal insulin + GLP-1RAs), GC gliflo-combo (basal insulin + gliflozin)

At the start of the extension study, the original 101 participants remained in the basal-bolus group, 90 participants were in the FRC group and 93 in the gliflo-combo group. At 24 months, there were additional 32 dropouts in the FRC group and 39 in the glifo-combo group (Fig. 1).

### Treatment effect

Fifty-five percent of patients completed the study in the two comparison arms. At 24 months, patients randomized to the three groups experienced non statistically different reductions in HbA1c level according to the intention-to-treat analysis (−0.8%, −0.7%, and −1.3%, fixed-ratio, gliflo-combo, and basal bolus, respectively). Similar results were also obtained in the per-protocol analysis (−1.2%, −1.2%, and −1.3%, respectively) (Table 2). In both analyses (intention-to-treat or per-protocol), the results obtained at 24 months were significantly different (*P* < 0.01) versus baseline.

**Table 2:** Results for the primary endpoint

Time (months)	0	6	12	24	Final HbA1c
Intention-to-treat					
BBI	8.5 ± 1.1 (*n* = 101)	−0.6 ± 0.8 (*n* = 101)	−1.1 ± 0.9 (*n* = 101)	−1.3 ± 1.0 (*n* = 101)	7.2 ± 0.8
FRC	8.5 ± 1.0 (*n* = 102)	−0.6 ± 0.8 (*n* = 102)	−0.7 ± 1.1 (*n* = 102)	−0.8 ± 1.1 (*n* = 102)	7.7 ± 0.9
Gliflo-combo	8.7 ± 1.1 (*n* = 102)	−0.7 ± 0.9 (*n* = 102)	−0.6 ± 0.9 (*n* = 102)	−0.8 ± 1.1 (*n* = 102)	7.9 ± 1.0

Per protocol					
BBI	8.5 ± 1.1	−0.6 ± 0,8	−1.1 ± 0.9	−1.3 ± 1.0	7.2 ± 0.8
	(*n* = 101)	(*n* = 101)	(*n* = 99)	(*n* = 98)	
FRC	8.5 ± 1.0	−0.6 ± 0.8	−1.0 ± 1.0	−1.2 ± 1.0	7.3 ± 0.9
	(*n* = 102)	(*n* = 90)	(*n* = 70)	(*n* = 58)	
Glifo-combo	8.7 ± 1.1	−0.7 ± 0.9	−0.8 ± 0.9	−1.2 ± 1.1	7.5 ± 1.0
	(*n* = 102)	(*n* = 93)	(*n* = 75)	(*n* = 54)	

Data are mean ± SD

*BBI* basal-bolus insulin, *FRC* fixed ratio combination of basal insulin + GLP-1RA, *gliflo-combo* basal insulin + a gliflozin pill (canagliflozin, empagliflozin, dapagliflozin)

The mean differences between FRC or gliflo-combo groups and the basal-bolus group were not statistically significant. The final HbA1c level (per protocol) was 7.2 [55 mmol/mol] ± 0.8%, 7.3 [56 mmol/mol] ± 0.9% and 7.5 [58 mmol/mol] ± 0.9%, respectively (*P* = NS).

The proportion of patients achieving HbA1c < 7.0% (53 mmol/mol) was 50%, 47%, and 45%, respectively (*P* = 0.456 for comparison among groups), and those achieving HbA1c < 7.5% (58 mmol/mol) was 62%, 60%, and 58%, respectively (*P* = 0.345) (Table 3).

**Table 3:** Proportion of patients with HbA1c ≤ 7% or ≤7.5%

Time (months)	0	6	12	24
*HbA1c* ≤ *7%*				
BBI	0%	34%	54%	62%
FRC	0%	28%	49%	60%
Gliflo-combo	0%	27%	46%	58%
* HbA1c* ≤ *7.5%*				
BBI	0%	19%	45%	50%
FRC	0%	19%	45%	47%
Gliflo-combo	0%	16%	41%	45%

*BBI* basal-bolus insulin, *FRC* fixed ratio combination of basal insulin + GLP-1RA, *gliflo-combo* basal insulin + a gliflozin pill (canagliflozin, empagliflozin, dapagliflozin). Per-protocol analysis

Compared with the 6-month data, total insulin increased in all groups at 24 months: BBI (from 62 U to 75 U, *P* < 0.05), FRC (from 27 U to 44 U, *P* < 0.01), and gliflo-combo (from 21 U to 45 U, *P* < 0.01). The number of daily injections remained unchanged, with four insulin injections daily in the basal-bolus group (one shot of basal insulin plus three shots of rapid insulin) and one injection daily in both FRC and gliflo-combo groups.

The mean dose of the GLP-1RA was 1.44 ± 1.2 mg liraglutide for IDegLira and 15 ± 7.5 µg lixisenatide for IGlarLixi.

Patients continuing the basal-bolus regimen exhibited significant amelioration in fasting plasma glucose associated with a significant increase in body weight. Patients in the FRC and gliflo-combo groups also showed significant improvements in fasting glucose but a significant reduction in body weight (Table 4). The baseline DTSQ scores were 16.3 ± 5.5, 17.4 ± 6.1, and 15.9 ± 5.8 in the three groups, respectively (basal-bolus, FRC, and gliflo-combo). At 24 months, DTSQ scores increased by 17.4 in the FRC and by 16.9 in the gliflo-combo groups, respectively, but remained unchanged in the basal-bolus group (Table 4).

**Table 4:** Results at 24 months

Variables	Basal-bolus insulin (*n* = 98)		Basal insulin + GLP-1RA (*n* = 58)		Basal insulin + gliflozin (*n* = 54)	
	Changes vs baseline	*P*	Changes vs baseline	*P*	Changes vs baseline	*P*
Fasting glucose, mg/dl	−35 ± 37	0.01	−33 ± 41	0.01	−29 ± 31	0.001
Weight, kg	1.8 ± 1.5	0.01	−2.2 ± 2.3	0.001	−0.9 ± 1.2	0.030
Daily insulin dose, units	25.3 ± 16.4	0.01	−4.1 ± 10.2	0.321	−3.8 ± 9.4	0.231
DTSQ	1.6 ± 3.1	0.245	17.4 ± 11.6	0.004	16.9 ± 9.1	0.001

Data are mean ± SD. The values of *P* indicate difference of changes from baseline. Per-protocol analysis

*DTSQ* Diabetes Treatment Satisfaction Questionnaire

There was no evidence of heterogeneity for predefined subgroups according to body mass index, age, gender, smoking status, and estimated glomerular filtration rate categories (data not shown).

### Adverse events

The proportion of patients presenting at least one episode of level 1hypoglycemia was 24.8%, 12.8%, and 9.9% in the basal-bolus, FRC, and gliflo-combo groups, respectively, with a significant difference among them (*P* = 0.011). Few patients (less than 7%) in the three groups experienced level 2 or 3 hypoglycemia.

There was no event of acute pancreatitis in the fixed-combo group. Five participants in the FCR group and seven participants in the gliflo-combo group discontinued for adverse events, mostly gastrointestinal in the FRC group and genital mycotic in the gliflo-combo group.

## Discussion

Our study is the first randomized trial to assess the durability of simplification of complex insulin regimens in patients with type 2 diabetes in their current clinical practice. In this setting, substituting a complex and ineffective full basal-bolus regimen for up to 24 months with the simpler strategy of either FRC or gliflo-combo achieved glucose control not significantly different from intensification of the previous basal-bolus. The added benefits of weight loss, less insulin doses, daily injections, and hypoglycemia, accompanied by increased patients’ satisfaction related to therapy modifications, were maintained in the extension period in at least one-half of the randomized patients.

Current treatment guidelines have a greater focus on intensification, rather than on simplification [9, 10]. The multitude of therapeutic options available makes treatment plans involving insulin unnecessarily complex for both clinicians and individuals with type 2 diabetes. The basal-bolus regimen still represents the best option in the current clinical practice at the top of type 2 diabetes management [9]. The results of BEYOND seem to confute the dogma about the inevitability and eternity of basal-bolus regimen in type 2 diabetes [11] by presenting the evidence around the feasibility and safety to switch either to a once-daily injection of a FRC or once-daily gliflozin pill added to basal insulin. This simplification strategy may work, in terms of significant and clinically relevant reduction of HbA1c, in at least one-half of patients who can maintain the benefits for 24 months.

The dropouts in both fixed-ratio and gliflo-combo groups were mainly due to inefficacy of maintaining the HbA1c level below the baseline value. Drop-out rates were significantly higher in both treatment groups than in the basal-bolus group which, by protocol, did not foresee drop-out. Future research should focus on how to predict future therapeutic ineffectiveness before switching from basal bolus to other therapeutic regimens. At present, statistical heterogeneity analysis suggests a lack of role for excess weight, duration of diabetes, smoking, age and renal function.

In conclusion, the simplification approach fails at 24 months in about one-half of patients with type 2 diabetes. All this is expected in the natural history of type 2 diabetes made up of therapeutic achievements and failures. On the other hand, the durable effects we observed in at least 50% of patients provide support for such an approach as an effective way to combat clinical inertia [12]. Simplifying therapeutic strategies, when suitable and without compromising treatment efficacy and safety, may offer the opportunity to ease disease burden in patients with type 2 diabetes.
